# Developing Integrated Extended Pharmacist Roles and Services for Equitable Access and Outcomes in Primary Healthcare: A Realist Evaluation

**DOI:** 10.34172/ijhpm.9127

**Published:** 2025-11-15

**Authors:** Tara N. Officer, Janet McDonald, Kirsten Smiler, Lynne Russell, Jonathan Kennedy, Eileen McKinlay, Marianna Churchward, Ausaga Fa’asalele Tanuvasa, Jacqueline Cumming, Mereana Pere, Mona Jeffreys, Caroline Morris

**Affiliations:** ^1^School of Nursing, Midwifery, and Health Practice, Victoria University of Wellington, Wellington, New Zealand.; ^2^Health Services Research Centre, Victoria University of Wellington, Wellington, New Zealand.; ^3^Department of Primary Health Care & General Practice, University of Otago, Wellington, New Zealand.; ^4^Centre for Interprofessional Education, Division of Health Sciences, University of Otago, Dunedin, New Zealand.

**Keywords:** Practice Pharmacist, Practice-Based Pharmacist, Primary Healthcare, General Practice, Aotearoa New Zealand, Realist Evaluation

## Abstract

**Background:**

Pharmacists’ roles have been moving from dispensing towards more patient-focused extended clinical care, signalling changes in health service policy and delivery. This paper evaluates the development of practice pharmacists’ roles and services in primary healthcare (PHC) settings in New Zealand (Aotearoa), how services were implemented, and how pharmacists, patients, and other PHC professionals responded to role and service developments.

**Methods:**

We applied a realist evaluation methodology, identifying context (C) into which initiatives are introduced, mechanism (M) triggered or attenuated by the context, and resulting outcome (O). CMO configurations were developed, tested and refined through rapid realist review, key informant interviews, six case studies, and a national survey. Although not every project stage is fully documented in this paper, each contributed to the final theories. The desired outcome was defined as: *Practice pharmacists are integrated within PHC teams, and their services are accepted and utilised by patients. Practice pharmacist services are equitably accessible throughout Aotearoa and contribute to equitable health outcomes*.

**Results:**

Four programme theories, based on 22 CMO configurations, showed how contexts and mechanisms at national, service, role, and patient levels could enable or attenuate reaching the desired outcome; for example, through a burning platform for change, supportive colleagues and infrastructure, well-fitting roles, and patient awareness, or conversely through, disjointed leadership, limited understanding and role uncertainty, and lack of access to practice pharmacists. Funding, regardless of source, operated across all levels as context and mechanism.

**Conclusion:**

Practice pharmacist services operate within multiple contexts and levels; their development and implementation requires a systems view. Opportunities exist to achieve the desired outcome by strengthening enabling contexts or reducing barriers within attenuating contexts. Policy-makers now need to focus on equitable service distribution, sustainable ways to fund and employ practice pharmacists, and the equity impact of patient copayments.

## Background

**Key Messages**

**Implications for policy makers**

- Pharmacists working in general practice (“practice pharmacists”) are a relatively new role that has developed in an ad hoc manner, but nonetheless, contributes to patient-focused care, practice-level activities (eg, medicines audit) and medicines information and education for other health professionals.
- Based on the New Zealand (Aotearoa) setting, this research identifies “what works” to develop the practice pharmacist role and implement services that are integrated within primary healthcare (PHC) teams, accepted and utilised by patients, equitably accessible and contribute to equitable outcomes.
- We identify multiple points where policy-makers can intervene to influence the likelihood of these desired outcomes – a systems view is essential.
- Key areas to focus on next are equitable service distribution, how best to fund and employ practice pharmacists, the impact on equity if patients pay for services, support for pharmacists’ post-graduate training, and a more diverse workforce.
- Addressing these areas poses a distinct challenge for policy-makers and planners as they manage competing service delivery demands, skill-mix concerns, and general health workforce shortages.

**Implications for the public**

Diversifying primary healthcare (PHC) workforce skill-mix can improve healthcare access and workforce retention, and potentially improve clinical outcomes. However, despite pharmacists increasingly adopting more direct patient care roles, including within general practice teams, there is limited knowledge about how these practice pharmacist roles support health service delivery. We found most patients did not fully know what pharmacists do, but when they received services, they developed understanding and trust in pharmacists’ skills. This understanding was developed also through other clinicians’ support of the role. Our research suggests that successful implementation of these roles requires a multi-pronged approach with partnership between policy-makers, established PHC clinicians, non-clinical employees, and pharmacists to support the embedding of these roles into clinical practice. A better understanding of how practice pharmacist role implementation and service delivery has occurred will allow government and clinical service providers to target resource investment in ways that more clearly supports equitable service delivery.

Pharmacists’ roles have shifted internationally from dispensing medicines to more patient-focused clinical roles in community pharmacy and primary healthcare (PHC).^1^ This paper explores the development of pharmacists’ roles in general practice, variously termed clinical pharmacists, general practice pharmacists, and practice or practice-based pharmacists (hereafter “practice pharmacists”). There has been significant investment in such roles in England, Scotland, and parts of Canada,^2-4^ with slower development in places such as the United States, Australia, and New Zealand (Aotearoa).^5-7^ Practice pharmacists undertake a wide variety of activities based on their pharmaceutical/pharmacology knowledge, including patient-focused care (eg, medicines education and management), practice-level activities (eg, medicine audits), and providing medicines information and education for other health professionals.^8,9^ Some practice pharmacists are also prescribers, with Great Britain moving towards all newly qualified pharmacists being independent prescribers once registered.^10,11^ These pharmacists work as members of healthcare teams; their roles may operate as complements and substitutes to others within the team to addresses clinical and other service needs across diverse populations.^12,13^

 Benefits of pharmacist roles include interprofessional collaboration in the PHC team, reduced medication-related problems and improved medicines adherence for patients, reduced prescribing errors, and general practice workload support and education.^2,14-17^ Some studies have also shown improved patient health outcomes, such as better hypertension and diabetes management,^14,18-20^ and that practice pharmacist services can be cost-effective.^21,22^ Patients have reported low initial awareness of practice pharmacist roles, but high acceptability of their services, along with improved understanding of their medicines and health conditions.^23-25^ Alongside these benefits, concerns about the roles primarily relate to funding barriers, sustainability, lack of trust, and policy and service fragmentation through having multiple providers.^5,26,27^

 In Aotearoa, practice pharmacist roles have developed unevenly around the country over roughly the last 10 years.^6^ Policy and professional documents over this time have emphasised the importance of health professionals working collaboratively in integrated healthcare teams, and the need to utilise the skills of the pharmacist workforce fully.^28-30^ This is partially in response to workforce pressures and a recognition of the importance of skill-mix changes (changes that may include, for example, task shifting, task sharing, role enhancement, or role substitution^12^) to manage increasing patient healthcare need complexity. The *New Zealand Health Strategy*^30^ prioritises creation of flexible, appropriate care provided through integrated health services utilising multi-disciplinary teams. The Strategy is underpinned by a commitment to Te Tiriti o Waitangi | the Treaty of Waitangi, a foundational document signed by Māori, the Indigenous people of Aotearoa, and the British Crown in 1840.^31^ The Strategy incorporates five principles drawn from Te Tiriti guaranteeing: tino rangatiratanga (Māori self-determination) and mana motuhake (the right for Māori to be Māori, to exercise authority over their lives, and to live on Māori terms and according to Māori philosophies, values and practices, including customary practices) in health and disability service design, delivery and monitoring; equity of health outcomes for Māori; active protection for Māori to achieve equitable health outcomes; options for health and disability services for Māori (including resourcing for kaupapa Māori services – services developed and implemented by Māori) that are provided in culturally appropriate ways that recognise and support expression of Māori healthcare models; and partnership between the Crown and Māori in the governance, design, delivery and monitoring of health and disability services, including the co-design of the PHC system for Māori.^30,32^ Consequently, these principles have direct implications for practice pharmacist role development in PHC, shaping expectations for equity, Māori health priorities, and culturally responsive service delivery.

 At the time the present research was undertaken, primary and secondary health services in Aotearoa were planned and funded through 20 district health boards (DHBs).^33^ Following 2022 health reforms, and further changes in 2024 by a new government, a single organisation, Health New Zealand | Te Whatu Ora (HNZ | TWO), now plans and funds services nationally.^34^ DHBs previously, and now HNZ | TWO, in turn fund 30 primary health organisations (PHOs) that coordinate PHC services for their enrolled populations, including contracting with general practices and other providers to deliver PHC services.^33,35^ General practices are mostly privately-owned and can charge patient fees in addition to the public funding they receive. Public funding and fees vary with patient demographic characteristics and whether they are eligible for a means-tested Community Services Card, which reduces some healthcare costs.^36,37^ Some general practices are eligible to receive additional funding if they agree to keep patient fees low; these Very Low Cost Access practices serve “high needs” populations (defined as having at least 50% of patients who are Māori, Pacific, or of low socioeconomic status).^38^

 Aotearoa pharmacists can register in the “pharmacist” or, with an additional qualification, “pharmacist prescriber” scope of practice. Practice pharmacists do not have a separate scope of practice, but the Pharmacy Council of New Zealand has a position statement about pharmacists in general practice, and there is a toolkit for pharmacists practising in this setting.^39,40^ In 2024, there were 94 pharmacists whose primary work setting was in general practice (2.3% of the total pharmacist workforce) and 32 (0.8%) working in a PHO.^41^ International research around the practice pharmacist role is often in countries where the role is more established or involving the evaluation of discrete pilot services.^5,42^ Our research presents an opportunity to understand how these roles form given growing changes towards practice-based roles for pharmacists.

 To date, there has been limited research into practice pharmacist roles and service development in Aotearoa; what exists has investigated the development of pharmacist prescriber roles, and briefly practice pharmacist integration into general practice and services provided.^6,43-46^ A survey of general practices with a practice pharmacist found practice pharmacists were viewed as adding significant value to clinical support, patient outcomes and patient safety, and to a lesser extent, support for general practitioner (GP) workload.^47^ Consequently, until now, there remains a dearth of knowledge around what has prevented greater development of the practice pharmacist role in Aotearoa.

 The research reported in the present paper formed part of a broader programme of work *Enhancing primary health care services to improve health in Aotearoa New Zealand* (five linked projects). The project described in this paper focussed on the period from late 2018 and sought to answer:

1. How is the practice pharmacist role expected to develop in Aotearoa in the next five years? How is development of the role expected to influence health and health system outcomes? What differences of views are there with respect to the development of the pharmacist role in Aotearoa?
2. How are current practice pharmacist services being implemented, nationally, at district level, and locally? What initiatives are practice pharmacists involved with to deliver extended pharmacy services? How are pharmacists, patients, and other PHC professionals responding? How are different contexts influencing the development of practice pharmacist role in Aotearoa?

## Methods

 Practice pharmacist services in Aotearoa are not implemented in a standard way nationwide but have evolved in various ways within a relatively permissive environment. They can be viewed as a complex intervention, involving interactions between multiple health service levels (national, regional and local) and multiple “actors” (including policy-makers, service planners and funders, varied health professionals and patients), being likely to be flexibly implemented according to local needs, and unlikely to work successfully in all contexts at all times. A theory-based approach to research can be used to explore complex interventions^48^; realist evaluation methodology^49^ was chosen for its focus on unpacking black boxes and identifying underlying generative causation within programmes and interventions.^49^

 Realist evaluation asks, “What works for whom in what circumstances, in what respects, and how?”^50^ (p. 3)Outcomes of an intervention will not be the same in all circumstances, but are contingent on mechanisms triggered in particular contexts.^49^ Contexts include political, social and organisational conditions, and can operate at multiple system levels.^51^ “Mechanism” is the realist term for how people interpret and respond to an initiative and its resources.^52^ Mechanisms depend on the context – some contexts trigger a mechanism to “fire” and generate a desired outcome, while in other contexts, the mechanism will be inhibited and the desired outcome will not result.^52^ A realist evaluation seeks to identify context-mechanism-outcome (CMO) configurations that are operating.^53^ In other words, realist evaluators are interested in understanding how an outcome occurred due to the relationship of context and mechanism. In this research, we explored contexts at five levels: national policy, regional service planning, PHC services, practice pharmacists, and patients. The desired outcomes from these services were drawn from policy documents^28,30,54,55^ and key informant interviews, ensuring they reflected the priorities and intentions of the policy environment. These outcomes served as reference points for CMO configuration development and testing. They are defined as follows: *Practice pharmacists are integrated within PHC teams, and their services are accepted and utilised by patients. Practice pharmacist services are equitably accessible throughout Aotearoa and contribute to equitable health outcomes*. This paper focusses on intermediate outcomes (the effective development and utilisation of practice pharmacist services) expected to improve health outcomes; more work will be needed to quantify the longer-term outcomes of practice pharmacist services.

###  Data Collection and Analysis

 This research was undertaken between October 2018 and March 2024. This encompassed the start of the COVID-19 pandemic, with a series of lockdowns in Aotearoa; a nationwide COVID-19 vaccination programme; and major health system reforms beginning in 2022, some of which were repealed in 2024 following a change of government. These changes contributed to significant, ongoing health service pressure (exacerbated by workforce shortages) and created opportunities to explore how different contexts influence practice pharmacist role and service development in turbulent environments. The research team (supported by a research advisory group that included pharmacy policy, commissioning, and sector representatives) undertook five largely sequential research phases (Table 1) as part of a mixed methods study design, with each informing the next.

**Table 1:** Research Phases and Data Sources

**Phase**	**Timeframe**	**Data Source**
Literature review	Ongoing throughout project	Review of key Aotearoa policy documents; ongoing national and international literature review throughout project. Conducted to situate the research and identify policy goals.
Rapid realist review	Oct 2018-Jul 2019	Focused search of literature (2008-2018) involving pharmacists working in primary (health) care and general practice using PubMed, Scopus, and Google Scholar databases and through expert panel sources/literature identified through a related project. Culminated in identification of 81 papers and development of “if-then” statements, which were tested and refined with two subject experts. Conducted to inform initial theories for testing.
Key informant interviews	May-Jul 2019	Interviews with seven key informants with roles in pharmacy policy, service contracting, education, practice, professional support, and a patient perspective. Conducted to inform initial theories.
Case studies	Sep 2020-Dec 2023	Interviews with practice pharmacists, other health professionals, patients, and local pharmacy service leads (42 participants in total). Conducted to further refine theories.
Survey	May-Aug 2022	Online survey for pharmacists practising in PHC settings; 39 practice pharmacist respondents (reported elsewhere^46^). Conducted as part of triangulation and understanding the context in which practice pharmacist roles develop.

Abbreviation: PHC, primary healthcare.

 Data reported in this paper came from the key informant interviews and subsequent case studies phases, with results also informed by literature and a rapid realist reviews, and our survey. In an earlier, related project about the development of extended pharmacist roles and services focused on community pharmacy, 50 key informants had been interviewed (including policy-makers, service planners and funders, pharmacists, other health professionals, and patients).^56^ These also provided background to practice pharmacist role development. For the project reported here, we undertook 7 further key informant interviews to deepen understanding of practice pharmacist role and service development. Key informants were selected because of their involvement in or knowledge of policy changes around developing practice pharmacist roles, informed by discussion with our advisory group. Informants were approached directly to request interviews; following obtaining written consent, interviews were conducted at a mutually agreed time and place. These interviews were coded for CMO configurations (See below).

 Originally, we planned to include up to eight in-depth case studies, focused on a practice pharmacist, other health professionals they worked with, patients, and pharmacy service funding and planning leads in the local area. We completed six case studies – recruitment was delayed and interrupted by the COVID-19 pandemic and associated lockdowns in Aotearoa, which also led to most interviews being undertaken by Zoom (Zoom Communications) or telephone, though in-person interviewing was possible for later cases. Cases reflected areas where practice pharmacist services were more and less developed; pharmacists based within a general practice or working from a PHO; differing funding arrangements; and a mix of patient populations, including Māori, Pacific, and low-income peoples.

 Recruitment for each case began with approaching a practice pharmacist and then, if they were interested in taking part, contacting a general practice owner or manager for consent for their practice to be a case study. Once this locality consent was given, arrangements were made to interview the pharmacist and they were asked to name other general practice team members and their local PHO and DHB pharmacy leads, whom the research team approached for interviews. The practice pharmacist was also provided with flyers about the research to give to patients, who could then contact the research team if they were interested in being interviewed. All participants gave informed consent before taking part. Patient and health professionals received a gift voucher as thanks for their participation.

 Interview schedules were informed by the prior rapid realist review and our key informant interviews. Topics for pharmacists included their workplace/s and employment arrangements, roles and services, the kinds of patients they worked with and patients’ responses to their role, relationships and integration with other staff and services, factors supporting or hindering the development and success of their role, and impacts of COVID-19. Interviews with other health professionals covered related topics, reframed as relevant for the participant’s perspective. Interviews with DHB and PHO pharmacy leads asked about regional pharmacy service planning, and the factors influencing practice pharmacist service development and sustainability. Patients were asked how they came to see a practice pharmacist, what services they had received, if and how these had helped them, and any changes they would like to make to these services. Participants were provided with the option for interview with Māori, Pacific or non-Māori/ non-Pacific interviewers. Interviews concluded with a short ranking exercise drawn from the literature, which provided opportunity to explore reasoning behind participants’ choices and identify the most important contexts and mechanisms that were operating. Interview schedules are available in Supplementary file 1.

**Figure 1:**
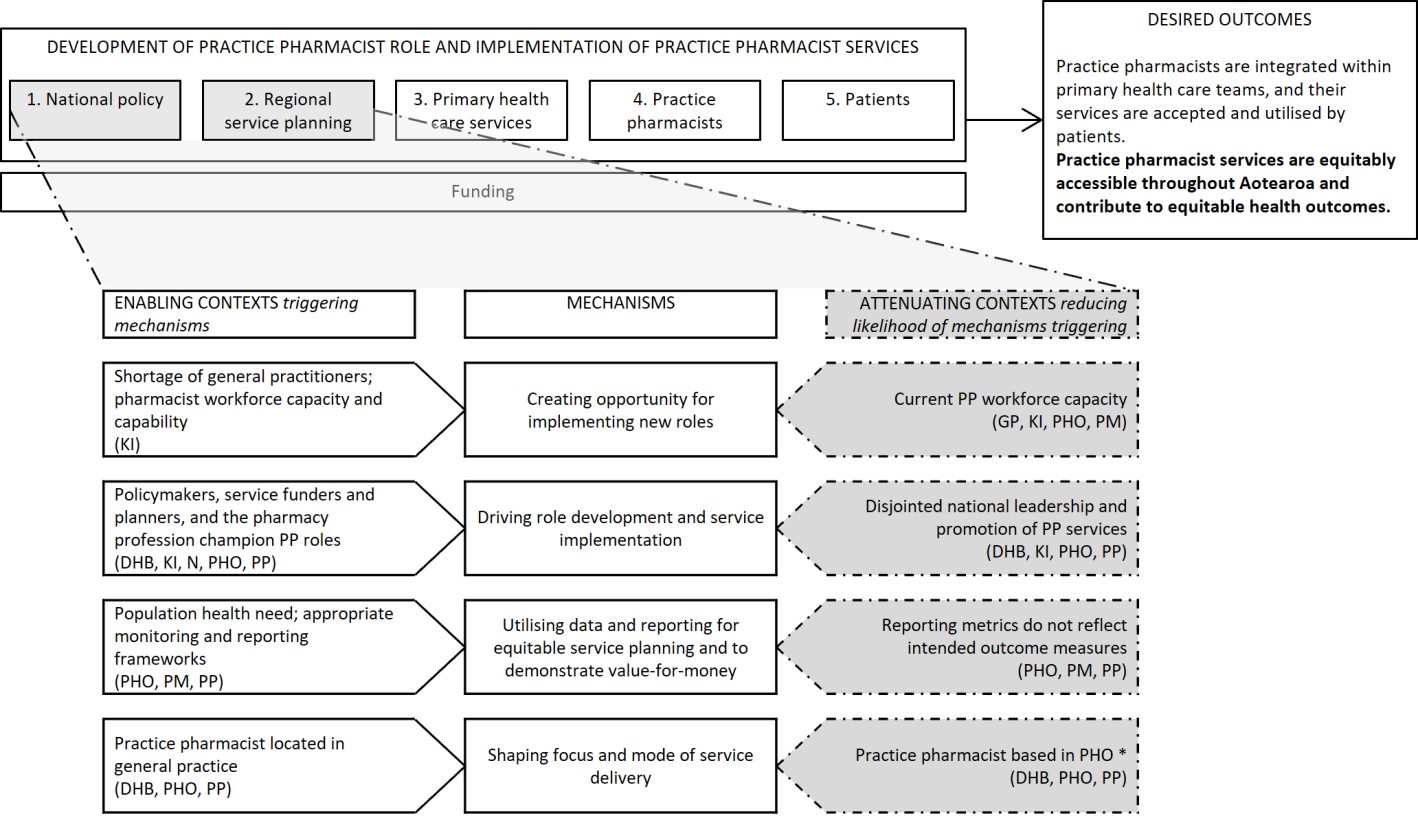
National Policy and Regional Service Planning Context-Mechanism-Outcome Configurations. Abbreviations: DHB, District Health Board (regional service funder and planner at the time of the research; now Health New Zealand | Te Whatu Ora); GP, general practitioner; KI,key informant; N, nurse; PHO, primary health organisation; PM, practice manager; PP, practice pharmacist. * Unlike other contexts in this figure, this context does not directly attenuate, instead it triggers the mechanism to deliver different sorts of practice pharmacist services.

**Figure 2:**
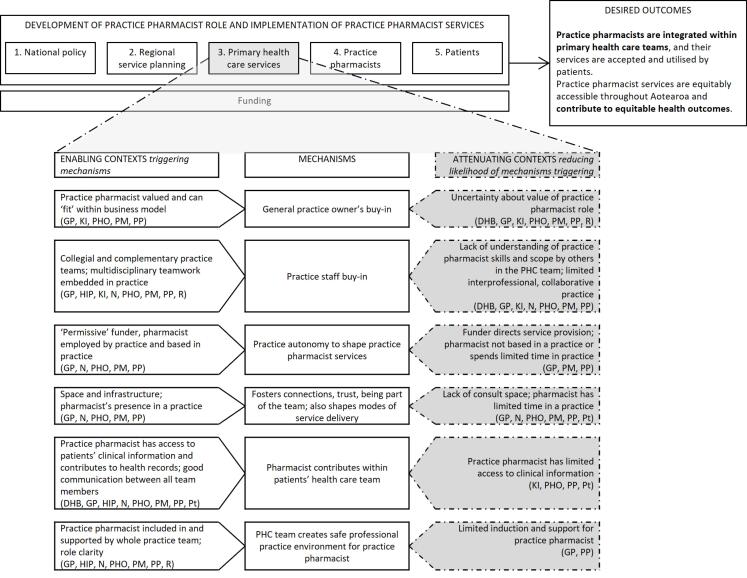
Primary Healthcare Services Context-Mechanism-Outcome Configurations. Abbreviations: DHB, District Health Board (regional service funder and planner at the time of the research; now Health New Zealand | Te Whatu Ora); GP, general practitioner; HIP, health improvement practitioner (registered health professionals based in general practice who provide brief interventions to patients, to improve well-being)^58^; KI, key informant; N, Nurse; PHO, primary health organisation; PM, Practice manager; PP, practice pharmacist; Pt, patient; R, receptionist; PHC, primary healthcare.

**Figure 3:**
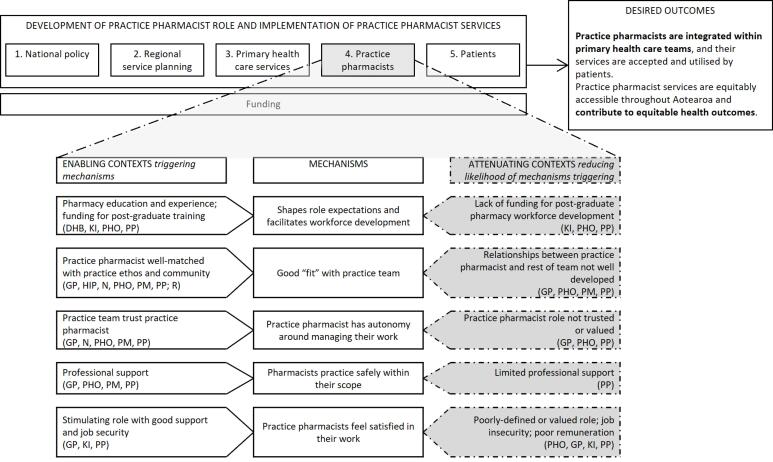
Practice Pharmacist Context-Mechanism-Outcome Configurations. Abbreviations: DHB, District Health Board (regional service funder and planner at the time of the research; now Health New Zealand | Te Whatu Ora); GP, general practitioner; HIP, health improvement practitioner (registered health professionals based in general practice who provide brief interventions to patients, to improve well-being)^58^; KI, key informant; N, Nurse; PHO, primary health organisation; PM, Practice manager; PP, practice pharmacist; R, receptionist.

**Figure 4:**
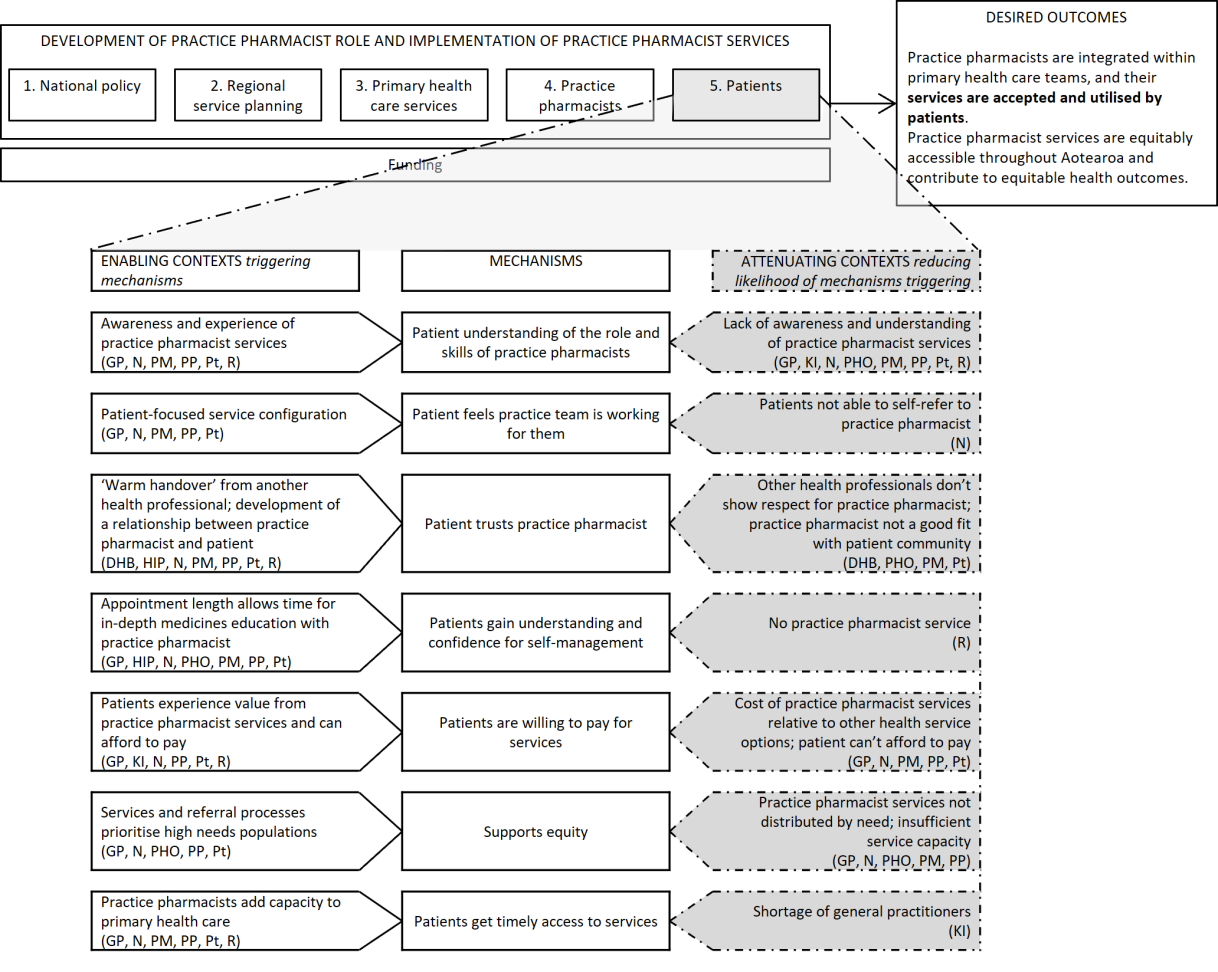
Patients Context-Mechanism-Outcome Configurations. Abbreviations: DHB, District Health Board (regional service funder and planner at the time of the research; now Health New Zealand | Te Whatu Ora); GP, general practitioner; HIP, health improvement practitioner (registered health professionals based in general practice who provide brief interventions to patients, to improve well-being)^58^; KI, key informant; N, Nurse; PHO, primary health organisation; PM, Practice manager; PP, practice pharmacist; Pt, patient; R, receptionist.

 With permission, all interviews were audio recorded and later transcribed. Participants could request a copy of their transcript and make changes if they wished. The interviews were initially coded thematically in NVivo data management software,^57^ using deductive codes derived from interview topics and inductive codes developed from additional ideas within the interviews. In a second step, quotes from interviews were linked with “if-then” statements. These built on statements developed from our earlier rapid realist review (and added new statements based on the interview data). In realist evaluation research, if-then statements indicate that *if *a mechanism operates in a particular context, *then* a specific outcome will occur.^53^ They present an easy way for a non-realist audience to identify levers of policy and practice change. These statements were discussed among the research team and revised, then continually added to and refined as interviewing continued. In this way, analysis of key informant interviews was added on top of/served to amend the findings of our review, which were then further amended in our case study phase. Summary tables grouped CMO configurations at five levels and these formed the basis of Figures 1, 2, 3 and 4. These levels were not pre-specified but reflected patterns and relationships emerging from the data. This enabled systematic exploration of how mechanisms operated within and across interconnected system layers and captured how changes at higher system levels shape and are shaped by service, practice pharmacist, and patient level changes. A selection of final CMO configurations was also presented to, and discussed with, the project’s research advisory group; their feedback was incorporated into further revisions. Based on these, we identified four core programme theories around practice pharmacist role development and service implementation.

## Results

 Six case studies (with seven general practices) were undertaken between September 2020 and December 2023. In five cases, the practice pharmacist was funded by the DHB or PHO (with two practices also contributing funding); one was funded by a general practice (that also charged patients for these services). One case was in a major city, three in regional cities and two in small, rural centres; five were Very Low Cost Access practices. Two practice pharmacists were prescribers. Table 2 lays out participants’ demography.

**Table 2:** Case Study Participants (n = 42)

**Participant**	**Gender**		**Age (y)**		**Ethnicity**^a^		**Years of Practice**
Practice pharmacist	Female	6	25-44	2	Māori Pacific NZ European Other	8	15 months-10 years in practice pharmacist role (median 4 years; average ~5 years)
	Male	1	45-64	4		1	
			65+	1		21	
						11	
Practice manager	Female	5	25-44	1			3.5-30 years in PHC
			45-64	3			
			65+	1			
GP	Female	3	25-44	2			2-45 years in PHC
	Male	3	45-64	3			
			65+	1			
Nurse	Female	7	25-44	2			1.5-25 years in PHC
			45-64	5			
Other practice staff	Female	3	45-64	3			~1.5-15 years in PHC
DHB and PHO pharmacy leads	Female	5	45-64	7			1-15 years in current role
	Male	2					
Patient	Female	5	45-64	4	Māori	5	Not applicable
	Male	2	65+	3	Pacific	1	
					NZ European	1	
					Other	1	

Abbreviations: PHC, primary healthcare; PHO, primary health organisation; DHB, district health board; GP, general practitioner; NZ, New Zealand. ^a^Six participants belonged to more than one ethnic group, so totals add to more than 42; health professional data have been combined to reduce identifiability.

 In the remainder of this section, the final programme theories are presented at four contextual levels: “national policy and regional service planning”; “PHC services”; “individual practice pharmacists”; and “patients,” reflecting the five levels identified earlier (and combining the first two). Key CMO configurations are summarised at each contextual level in Figures 1-4: enabling contexts shown on the left are likely to trigger the central mechanism, while attenuating contexts shown on the right reduce the likelihood of the mechanism being triggered (indicated by the dashed arrow). By distinguishing enabling or attenuating contexts, we have identified specific challenges and leverage opportunities, providing clear direction for optimising role development and integration within PHC. The aspects of the outcome to which each group of CMO configurations contribute are bolded in the desired outcome. Note that multiple contexts operate simultaneously and across the different levels and can thus produce varied outcome patterns. The text explains each diagram and provides illustrative interview quotes. Readers interested in specific programme theories can refer to corresponding sections of the discussion where these are revisited.

###  Programme Theory 1: National Policy and Regional Service Planning

Figure 1 sets out four CMO configurations operating within national policy and regional service planning. At this level, consideration is given to the role of high-level actors and regional planning influencing practice pharmacist role development and service implementation. The overarching theory guiding this level is:

 If there is a “burning platform” for change and the value of practice pharmacists is recognised by policy-makers and service funders and planners (C), then this creates opportunity and drive (M) for role development and service implementation (O). If funders focus on population health needs (C), they will prioritise (M) equitable access to practice pharmacist services (O). Locating practice pharmacists within general practice or at a PHO (C) shapes the ease with which relationships and trust can be formed with general practice teams (M).

 A key informant suggested:

 “*The drivers for where we see the [practice pharmacist] role being integrated and developed are around understanding that primary health care teams are under pressure, and we’ve got an ageing general practice workforce… [Practice pharmacists] are an added skill set… to support these people with higher and more complex needs to live well and stay well in communities” *(KI3).

 Similarly, health policy documents and successive Health Ministers frequently refer to health system pressures arising from an ageing population and a growing prevalence of long-term conditions. They also highlight the opportunity to utilise pharmacist capacity and expertise to support PHC in addressing these challenges.^28,30^ Together, these form part of the context for a burning platform, ie, one where there is urgency and a compelling rationale for change. However, the current practice pharmacist workforce is small, and participants noted recruitment in rural areas may be difficult. Opportunities for upskilling may also be restricted:

 “*There’s also limited spaces in those tertiary facilities to be able to support… [practice pharmacists to train as] prescribers”*(Case5PHO1).

 Leadership from the pharmacy profession, policy-makers, and service funders and planners was identified as critical to developing practice pharmacist roles and service implementation. Some participants thought there needed to be stronger national championing of practice pharmacists:

 “*There isn’t a coordinated view from the [pharmacy] profession and there isn’t a single voice to the policymakers and subsequently the funders... Our value proposition is not necessarily clearly understood by policymakers and so, therefore, it’s difficult to be part of the conversation about redesigning care” *(KI6).

 “*As for promotion of the clinical pharmacist’s role in general, maybe that should be a national thing… It should be more out there of what they are, what they do and the qualifications that they’ve got” *(Case1PHO1).

 Some participants said that if then DHBs (now districts of HNZ | TWO) and PHOs have appropriate health data about their populations, then this can be used to design and monitor equitable services and demonstrate value. While one PHO participant viewed this as straightforward (monitoring such thing as caseloads, polypharmacy trends, equity of outcomes, and value for money), another described challenges in measuring value because some outcomes, such as impact within a multi-disciplinary team *“you can’t really measure with numbers… We haven’t quite… nailed the reporting”* (Case5PHO1).

 All pharmacists in this study spent at least part of their time working at general practice sites. Some were fully embedded in one or more general practice/s, while others were based at PHO offices. Funding availability influenced where practice pharmacists were placed, and their location influenced the service model:

 “*In the PHO environment it tends to be focusing on the population health and… [actioning] guidelines. You go and do audits and then you tell people, ‘Here’s the information.’ If you’re working within the practice, you actually really are part of the team. And it tends to be a lot more individual [patient-focused]” *(Case5PP1).

###  Programme Theory 2: Primary Healthcare Services

Figure 2 shows six CMO configurations operating at PHC services level. At this level, consideration was given to how general practices/PHOs, including staff, owners, and practice pharmacists worked together to form a PHC team. The programme theory formed from these CMOs is:

 If practice pharmacists are valued and supported within PHC teams and have appropriate resources (C), then they will be welcomed and can practise safely (M). Funder requirements, where and how practice pharmacists are based and employed, practice infrastructure and the time a practice pharmacist spends at a practice (C) influence services practice pharmacist offer and how they are delivered (M). The optimal outcome is practice pharmacists working collaboratively with PHC teams, delivering integrated services.

 When practice pharmacists’ skills and what they could offer were recognised and valued by others in a general practice team, this promoted general practice owners and other staff’s acceptance of new roles and services. Conversely, lack of understanding or valuing of practice pharmacist roles impeded uptake, but seeing a practice pharmacist at work could shift attitudes and encourage role acceptance.

 “*A lot of the medical profession are now seeing the value that pharmacists can make and respecting them” *(KI1).

 “*Coming from someone who doesn’t know much about primary care, when I found out about [pharmacist’s] role, I was like, ‘I don’t really understand what you do. Like I don’t see the point of this.’ I think that can be a bit of a barrier, because now that I have worked with a clinical pharmacist, I couldn’t imagine not having one around” *(Case5N2).

 The Health Care Home model of practice (based on the American Patient-Centered Medical Home model) has been developing in Aotearoa since about 2010.^59,60^ The model has a focus on an extended practice team,^59,60^ and participants believed it contributed to enabling contexts for welcoming practice pharmacists. Being included in and supported by the practice team was important for ensuring practice pharmacists worked safely.

 When practice pharmacists were employed by a general practice, the practice had autonomy to shape services to fit practice and patient needs best. When the practice pharmacist was externally funded and employed (by a PHO/DHB), then the funder may influence how the pharmacist was utilised, as illustrated by two practices with contrasting experiences:

 “*The PHO, the buck stops with their funding. They fund the person and then… we’ve pretty much run it how we want it run” *(Case1PM1).

 “*Our pharmacist is employed by our PHO… [Over time, there have been changes in] how she’s utilised because it’s often directed by her employer… I would love to be able to have [the pharmacist]… employed by us… How she’s contracted and who’s funded her, obviously that will set the foundation for how she could be utilised … [and] then it also falls into where she will be based” *(Case4PM1).

 On-site presence of a pharmacist was important fordeveloping relationships and trust between practice team members (through formal and informal interactions), and these took longer to develop when pharmacists were not co-located: *“I couldn’t imagine our clinical pharmacist being as successful or doing the role that she is doing if she wasn’t on site… If she was an email person or someone that we just know of, that relationship doesn’t develop, and therefore [the role’s] not used” *(Case5N2). The amount of time practice pharmacists spent in a general practice, and particularly being able to undertake patient consultations, was influenced by available space. Participants noted lack of space or being off-site shaped what services could be offered (particularly patient consultations) and how they were delivered.

 COVID-19 restrictions forced health services to change their ways of working, particularly the move to telehealth consultations. Some practice pharmacists have subsequently continued to offer *“virtual consultations… so that’s another way of access”* (Case5PHO1), though this service model was not appropriate for all patients: *“[During COVID] we wanted to talk to them virtually when a lot of our people wanted to come in. English as a second language is difficult when you’re on the telephone”* (Case5PP1).

 Practice pharmacists needed access to patients’ clinical information and in turn, to be able to contribute to their health records and communicate with others in the team, to ensure effective patient care. All had on-site access to patient management systems to facilitate this. Some participants contrasted this with community pharmacists who did not have access to the same clinical information about patients or ease of communication with other PHC professionals. For example, a GP commented, *“If you’ve got the pharmacist within the practice, you’ve got integrated care. If you’ve got them outside of the practice, you’ve fragmented it… You’ve not got the shared notes. You don’t have the longevity of the relationship”* (Case5GP1).

###  Programme Theory 3: Practice Pharmacists

Figure 3 sets out five CMO configurations; at this level, we have considered individual practice pharmacist’s roles and the contexts and mechanisms influencing their trajectories. The theory built around this follows:

 Undergraduate pharmacy education (C) shapes pharmacists’ role expectations (M) and funding availability for post-graduate training (C) facilitates practice pharmacist workforce development (M). If practice pharmacists are well-matched with a general practice ethos and its community, are trusted by other team members, have good professional support, and attractive work conditions (C), then they will find practice is safe and satisfying (M). When practice pharmacist services work well, integration and collaboration within PHC will be strengthened (O).

 Undergraduate pharmacy education is important in shaping pharmacists’ role expectations and practice preparedness,^61^ with a key informant stating that the Schools of Pharmacy (three in Aotearoa) need to *“consistently graduate people with the skills that are appropriate for that level of practice”* (KI7). Another said, *“I think the universities do have a role to play in terms of influencing and providing the evidence to support change and also for producing change makers as well”* (KI6). The Pharmacy Council of New Zealand position statement about practice pharmacists includes expectation of a pharmacy post-graduate certificate.^39^ HNZ | TWO provides national funding for postgraduate training for some health professionals (particularly doctors and nurses). Until very recently, when some funding for pharmacist prescriber training has become available,^62^ pharmacists have generally had to self-fund higher studies unless they receive local support by, for example, their PHO. Some participants considered lack of post-graduate funding for pharmacists as a barrier to study and entry into practice pharmacist roles. The need for more pharmacist workforce diversity was also noted:

 “*It would be really good if we could support Māori and Pacific pharmacists… We talk about having a diverse workforce to match our populations… it would be really amazing if we could have a workforce that matched the populations that they’re serving” *(Case5PHO1).

 If a pharmacist was well-matched with a general practice and its community, then this created a sense of “fit,” which supported teamwork and patient acceptance of practice pharmacist services, while conversely, *“You would learn pretty quickly if they didn’t fit in because the community just would not want to come. They would not engage”* (Case1PM1).

 When other health professionals trusted practice pharmacist professional competence, this enabled pharmacists to practise autonomously, particularly regarding prescribing, for those pharmacists who had this practice scope. For non-prescriber pharmacists, being trusted was also reflected in having their recommendations acted on by prescribers.

 In addition to collegial support within practice teams, pharmacists needed support from professional peers for professional development and safe practice. Well-defined roles, job security, and appropriate remuneration were also important for practice pharmacists’ work satisfaction.

 “*[Practice pharmacist] is the only one on a permanent role. The rest are on fixed-term agreements because none of our funding is permanent… It’s incredibly hard to recruit and retain good people… when you’ve got roles temporary in nature” *(Case2PHO1).

###  Programme Theory 4: Patients

Figure 4 sets out seven CMO configurations relating to patients’ practice pharmacist service experience. These were developed from the seven patient interviews and applying comments from other participants who provided commentary on patients’ experiences with these services. Patient voices have been prioritised in the quotes below. These CMOs focus on practice pharmacist service implementation, and how patients view such services. The programme theory formed from these CMOs is:

 If patients have positive experiences of practice pharmacist services (C), then they will develop understanding and trust in practice pharmacist roles and skills (M). A patient-focused service configuration and endorsement of other health professionals (C) also engenders patient receptivity and trust (M) in practice pharmacist services. When appointment length allows time for in-depth medicines education (C), then patients increase their understanding of their condition and medication, and gain confidence in self-management (M). Having experienced value from practice pharmacist services (and assuming patients can afford to pay) (C), then patients may be willing to pay for services (M). If services and referral processes prioritise high needs populations (C), then this supports equity of access for patients (M). If practice pharmacist services add capacity to PHC (C), then patients may receive more timely access to services (M). These configurations contribute to patients’ acceptance and utilisation of practice pharmacist services (O).

 Most patients did not understand the practice pharmacist role beforehand but having received services, they gained appreciation of practice pharmacists’ skills: *“Now I’ve seen what she does, I can understand how she can help”* (Case2Pt2). Greater promotion of these services, especially for high-needs communities, could support patient uptake: “*I don’t think a lot of people know about that role that much… especially us [Pacific communities]… I know for a fact that a lot of our old people need that services… and they won’t know unless [pharmacists] come to them”* (Case5Pt1).

 A patient-focused service configuration, considering aspects including location and time availability of services, and demonstrated collaboration and communication between health professionals, engendered patients’ sense that healthcare teams were *“all working together for the same results”* (Case2Pt1) and *“involving you, the patient, in discussions, decisions about your health”* (Case6Pt1).

 Having other practice staff connect patients with a practice pharmacist and endorse their work, and patients having time to form a relationship with the pharmacist, supported the development of patients’ trust in the practice pharmacist’s role. The following quotation illustrates the importance of culturally safe care for a patient:

 “*She’s culturally sensitive to me as a Māori… If I didn’t feel comfortable with her and knowing that she can affect my wellbeing et cetera, I would shut it down and be uncommunicative… But I felt confident with her; she gave me that feeling” *(Case2Pt2).

 Many of the patients spoke about being able to spend extended time with the practice pharmacist, which allowed for in-depth explanation of health conditions and medications, resulting in greater patient understanding and confidence.

 Practice pharmacist employment and funding arrangements affected whether patients paid for pharmacist appointments. Those with DHB/PHO funding did not charge for appointments; nevertheless, if patients experienced value from practice pharmacist services, then they could be willing to pay: *“I was ready to get my wallet out, thought I was going to have to pay… Wouldn’t worry me [to pay] if she’s getting the information I want; I would be quite happy to pay”* (Case2Pt3). However, if patients could not afford to pay, a fee for service would be a barrier to access: *“Our low socio – [economic population], it would impact them in that they wouldn’t go”* (Case2Pt2).

 Practice pharmacists operated in a variety of practice settings, of varying sizes and staffing capacity, and delivered services to different populations. For example, some were dedicated to caring for specific conditions, others managed complex patient care, and still others delivered services based on the introduction of new services, medicines, or audit results. Within these roles, prioritising practice pharmacist services for high needs populations supported equity, while failure to do so appeared to widen inequity.

 “*There was a medication that came on the market for diabetes, and it really had some real benefits and long term gains particularly for Māori and Pacific, so we put in some funding to support clinical pharmacists to support consultations, and we’ve seen real change in terms of the uptake, and in terms of equity. They played a real part in engaging in those priority populations and explaining what the new medication was about” *(Case5PHO1).

 In highlighting equity implications of the role, where a practice pharmacist added capacity to a general practice, then patients were seen as gaining more timely service access.

 “*Generally, everybody’s very open to [having an appointment with a practice pharmacist] and very appreciative, I think mainly because otherwise they would have to wait for one to two weeks to get to see their particular GP” *(Case3PP1).

###  The Role of Funding

 Funding or payment for practice pharmacist services featured at all levels of practice pharmacist role development and service implementation, from national to individual patient levels. This could take the form of funding limitations, district and government funding priority setting, and out-of-pocket payments. Regardless, as a concept, funding operated as a context and mechanism. As a context, if external funding was available to general practice for practice pharmacist services (C), then practices were incentivised to “buy in” to developing services (M). If practices commit funding themselves (C), then they had *“skin in the game”* (Case3DHB1) and were incentivised to ensure services’ success (M). The funding context also impacted whether patients paid for services:

 “*We have a mixed model within the practices, so for those practices that self-fund, then they have the ability to charge that to patients because they are self-funding it. Whereas the ones who are fully funded, they don’t have the ability to do that, which is fair and reasonable” *(Case5PHO1).

 Funding could also be a mechanism (in this case, a resource for practice pharmacist service development and implementation). If national or regional funders, or general practice businesses, valued practice pharmacist services (C), then they may be willing to fund them (M), with rationing and prioritisation when available funding is limited. However, the quote below illustrates that funding alone was insufficient to produce the desired outcome; other CMO configurations are also at play:

 “[*The practice pharmacist is] somebody that comes, they create an issue in that we have to find room for them, and we have to scrabble to create work for them… I mean we didn’t actively seek this role; [the PHO] thrust it upon us. If it cost us money, we wouldn’t buy it” *(Case1GP1).

## Discussion

 Four programme theories have been presented, summarised in Figures 1-4. The discussion, now, revisits each programme theory in turn, examining its broader implications. Although CMO configurations have been set out at four levels, practice pharmacist services and their role development operate at all levels simultaneously and each contributes to whether the desired outcome is achieved. Services operate within multiple contexts, some enable, while others attenuate mechanisms generating the desired outcome. Therefore, a systems focus, with recognition of the linkage of mechanism with enabling or attenuating context, is essential when developing and implementing such services. Many CMO configuration combinations are possible and achieving the desired outcome will depend on relative strengths of these contexts. Multiple opportunities exist to intervene to increase the likelihood of achieving the desired outcome by strengthening enabling contexts or reducing attenuating contexts. This discussion highlights key contexts on which to focus to optimise outcome delivery. In this way, our paper contributes to extant knowledge around the practice pharmacist role by emphasising key points impacting development of these roles in Aotearoa to date and opportunities for actionable change.

###  National Policy and Regional Service Planning

 Practice pharmacist services have evolved in an ad hoc fashion in many countries, enabling development of an innovative role but also resulting in uneven geographical service spread, varied funding and employment patterns, and differing ability to support equitable health outcomes. Recent additional primary and community health team funding in Aotearoa (which can include practice pharmacists) focussed on equitable access to PHC and prioritised Māori, Pacific and rural populations.^63^ Importantly, there remains a need to ensure achievement of policy aims of equitable service access and health outcomes (underpinned by Te Tiriti o Waitangi obligations to ensure equity of health outcomes for Māori, and active protection to ensure this).^28,30^ The funding, brought in under the previous government, concluded in June 2025 and the incumbent government has spoken of reviewing this scheme before deciding what will be continued.^64^ The formation in 2022 of a single, national funding and planning organisation, replacing 20 DHBs, may be one route to facilitate equitable practice pharmacist service distribution. Notably, returning to our desired outcomes, the ability to ascribe equitable outcomes to the practice pharmacist role is, therefore, impaired in part due to the ad hoc role development (a situation not unique to Aotearoa). Without consistent policy, diverse local models may remain fragmented, limiting their growth, durability, and equity. Further work is required to identify what clinical outcomes or end points are intended of practice pharmacists.

###  Primary Healthcare Services

 Our research highlights the importance of practice pharmacist employers and service location. Embedding practice pharmacists within general practices (rather than being based in a regional or coordinating health organisation) can more readily foster connections, trust, and communication with other PHC staff, and more interprofessional collaboration.^65-68^ In these situations, practices had autonomy to shape their practice pharmacist services, which was important to role development and service implementation. This was influenced by the degree of control a practice had in funding and employing the pharmacist, or an external funder’s degree of directiveness versus permissiveness in how a service could be organised. Similar to our research, a study comparing two models of funding and employing practice pharmacists in England (employed and embedded within general practices or externally employed and working across practices) found a key difference was the amount of control practices had to shape what the pharmacist did^69^; similarly Welsh pharmacists who had worked both in clusters of practices and a single practice said being employed by a practice supported their integration in the practice.^70^

 When setting up new services, practical issues, common across all settings, such as office space and layout, a clear job description, good induction, and ongoing support for practice pharmacists are important for facilitating safe working environments and collaborative teamwork.^5,40,71^ Consultation space and the time practice pharmacists spend at a practice also shape service offerings, particularly consultations with patients. Contexts triggering mechanisms of practice staff buy-in to what practice pharmacists can offer are more likely to lead to integration of practice pharmacist services within PHC teams. This was evident in cases that had inclusive workplace cultures and existing interprofessional models of practice. Conversely, lack of understanding of practice pharmacists’ skills or rigid professional boundaries generated initial resistance to accepting a new role. However, this could be overcome in time as staff observe what a practice pharmacist does and experience benefits from their contribution, as has been noted elsewhere.^3,66^ A “champion” within a practice can promote the role and encourage buy-in.^44,66^ This illustrates that contexts are not fixed and practices can be influenced to move from contexts “likely to attenuate,” to “likely to enable” mechanism triggering.

###  Practice Pharmacists

 Against a global backdrop of increasing role expansion, practice skill-mix changes, and consideration of roles as substitutes/complements, it is particularly notable that practice pharmacists, despite the wide variety of roles and recognised specialisation, do not operate within a separate scope of practice. In Aotearoa, the practice pharmacist role is encompassed within the “pharmacist” scope of practice, although a post-graduate qualification is often expected, and pharmacist prescribers must complete an accredited post-graduate training course.^39,72^ Most pharmacists have had to self-fund post-graduate studies, which presents barriers to training; the sector has welcomed some recent funding from HNZ | TWO (available until 2026) for pharmacists to train as prescribers.^62^ However, given pharmacists need to fund prerequisite qualifications, such an incentive will not offer a full pathway to pharmacist prescribing. Put alongside developments in the United Kingdom for undergraduate pharmacist prescribing teaching,^73^ it is timely to consider also pedagogical approaches for training practice pharmacists and the expected skill requirements for people in these roles. In turn, existence of advanced or formalised scopes of practice in some countries demonstrates a pathway for expanded roles to be formally recognised and embedded within health systems.^74^

 The *New Zealand Health Strategy *recognises the need for a diverse and representative health workforce as a contributor to achieving equitable outcomes.^30^ The current proportion of pharmacists identifying as Māori (2.5%) or Pacific (2.16%) is much lower than their population proportions (17.8% and 8.9% respectively),^41,75^ and continuing policies to support Māori and Pacific students at Aotearoa Schools of Pharmacy is essential.^76,77^ Further, previous studies have described some lack of understanding of practice pharmacists’ skills and roles or resistance to their role among other general practice staff, which can be overcome by working together and experiencing the contribution pharmacists bring.^5,43,78,79^ Interprofessional education at pre- and post-registration levels also plays key roles in developing collaborative practice.^80,81^ Together, these approaches may ensure workforce representation and skill recognition.

 Pharmacists have reported job satisfaction from patient-focused practice roles that utilise their clinical skills.^43,67,82^ In contrast, an exploratory study in Australia highlighted part-time employment and the role not being fully utilised as reasons practice pharmacists left their jobs and suggested clear role descriptions were important for retention.^83^ The findings of the current study suggest job security and remuneration will contribute to pharmacists’ satisfaction. Consideration also needs to be given to the impact of developing the practice pharmacist workforce on other groups in the profession, such as community or hospital pharmacy, so that workforce shortages are not inadvertently created elsewhere.^84^

###  Patients

 Patients who have experienced practice pharmacist services have reported high satisfaction, yet many patients are unfamiliar with these pharmacists’ roles and skills.^85-87^ Aotearoa research on advanced practitioner roles, including the pharmacist prescriber roles, highlighted patient perceptions that advanced practitioners act as a less optimal alternative to doctors operating within embedded structural hierarchies.^88^ Yet, patients also recognise and appreciate that advanced practitioners operate differently from GPs, delivering care that facilitates discussion and enabling patients to feel known.^89^ Greater role promotion and the support of other health professionals would increase patient awareness and willingness to utilise these services.^23,90^ Arguably, such promotion is difficult without having a defined practice scope in place, or system transformation facilitating health professional recognition of the value of other roles.

 International experience highlights how funding models for practice pharmacist services may influence patient access and role sustainability.^91,92^ A key consideration, therefore, becomes whether patients pay for practice pharmacist services and how this impacts equitable access. If there is no government funding for these services, general practices (most of which are private businesses in Aotearoa) can charge fees to cover costs. Services will then be less likely to develop in areas where patients cannot afford to pay, yet these are also likely to be high need areas.^93^ HNZ | TWO now plans and funds services nationally and is guided by the *New Zealand Health Strategy *with commitment to actioning obligations to Māori under Te Tiriti o Waitangi, including active protection for Māori to achieve equitable health outcomes.^30^ Equity should, therefore, be an essential focus when allocating practice pharmacist services funding, along with ensuring funding enables sustainable services.^5,43,66^ These variations, documented internationally and domestically, suggest that payment structures are not merely administrative detail, but active determinants of equity, shaping who benefits from practice pharmacist care and how these roles become embedded within health systems.

###  Strengths and Limitations

 By applying realist evaluation methodology, this research has created four programme theories that explain why practice pharmacist roles and their extended services are likely to produce a desired outcome in some PHC settings and less so in others. This work, therefore, offers an evidence base and starting point for developing a practical framework for policy-makers, service planners, and professional bodies to strengthen role uptake, integration, and equity in PHC locally and internationally. Previous realist evaluations have framed the achievement of an outcome around “enabling and constraining factors” that mean a mechanism is more likely (or less so) to produce a particular outcome,^94^ or used the “dimmer switch” analogy to indicate that a context activating a mechanism occurs along a continuum^95^; this research builds on these ideas and contributes the concept of enabling and attenuating contexts. In recognising that a switch may do more than dim, but rather change in some way the effect of an enabling context, the present research recognises that where there are large programmes under study, it is important to consider the dimmer switch continuum as occurring at two places, within an individual positive CMO configuration and directly against the configuration.

 The findings are drawn from six diverse cases that included multiple perspectives (funders and planners, pharmacists, other health professionals and practice staff, and patients who had used services, from varying ethnicities). The contributions of each group are noted in Figures 1-4. Some of these figures build on data from large and varied perspectives, enabling creation of refined configurations and the identification of particular groups to work with in addressing an enabling context or reducing the impact of attenuating contexts. Other CMO configurations are built on fewer participant perspectives, highlighting areas where further refinement is needed. COVID-19 interruptions impeded recruitment; in particular, a broader range of patients (including more Pacific participants and some who had not experienced practice pharmacist services) would strengthen the findings. The findings of any realist evaluation remain provisional and open to further testing and refinement.^96^ Future research in Aotearoa and internationally can strengthen and challenge theories presented here and apply or revise them to fit local contexts. In addition, this research focused on intermediate outcomes (the effective development and utilisation of practice pharmacist services) rather than clinical or health outcomes from these services; further work is needed to quantify longer-term outcomes and build on existing evidence of the effectiveness of these roles and services.^19-22^

## Conclusions

 Practice pharmacist services operate within multiple contexts at different levels, necessitating a systems view when developing and implementing these services. In future, as practice pharmacist services are embedded in Aotearoa and worldwide, key points to consider include equitable service distribution; the impact on equity if patients have to pay for services; how best to fund and employ practice pharmacists; and support for pharmacists’ post-graduate training and a more diverse workforce.

## Acknowledgements

 The authors would like to thank all the research participants, the members of the research advisory group and our colleagues in the wider programme grant of which this project formed a part. In particular, we acknowledge the support of Claire O’Loughlin, Lesley Middleton and Phoebe Dunn early in the project.

## Disclosure of artificial intelligence (AI) use

 Not applicable.

## Ethical issues

 All phases of the research had ethical approval granted by the Human Ethics Committee of Te Herenga Waka–Victoria University of Wellington: key informant interviews (#23937), case study interviews (#28419), and survey of pharmacists (#30080).

## Conflicts of interest

 Authors declare that they have no conflicts of interest.

## Supplementary files


Supplementary file 1. Interview Schedules.
