# Supplementary material for: Developing Integrated Extended Pharmacist Roles and Services for Equitable Access and Outcomes in Primary Healthcare: A Realist Evaluation
Source: Int J Health Policy Manag. 2025 Nov 15;14:9127. doi: 10.34172/ijhpm.9127 (PMC12958236; doi:10.34172/ijhpm.9127)
Supplement: Supplementary file 1 — Interview Schedules. [file ijhpm-14-9127-s001.pdf]

Article title: Developing Integrated Extended Pharmacist Roles and Services for Equitable Access and Outcomes in Primary Healthcare: A Realist Evaluation

Journal name: International Journal of Health Policy and Management (IJHPM)

Authors' information: Tara N. Officer[*1], Janet McDonald[2], Kirsten Smiler[2], Lynne Russell[2], Jonathan Kennedy[3], Eileen McKinlay[4], Marianna Churchward[2], Ausaga Fa'asalele Tanuvasa[2], Jacqueline Cumming[2], Mereana Pere[2], Mona Jeffreys[2], Caroline Morris[3]

[1]School of Nursing, Midwifery, and Health Practice, Victoria University of Wellington, Wellington, New Zealand.

[2]Health Services Research Centre, Victoria University of Wellington, Wellington, New Zealand.

[3]Department of Primary Health Care & General Practice, University of Otago, Wellington, New Zealand.

[4]Centre for Interprofessional Education, Division of Health Sciences, University of Otago, Dunedin, New Zealand.

***Correspondence to:** Tara N. Officer; Email: tara.officer@vuw.ac.nz

Citation: Officer TN, McDonald J, Smiler K, et al. Developing integrated extended pharmacist roles and services for equitable access and outcomes in primary healthcare: a realist evaluation. Int J Health Policy Manag. 2025;14:9127. doi: 10.34172/ijhpm.9127

Supplementary file 1. Interview Schedules

.A. Key informant interviews

Expanding the Role of Primary Health Care Pharmacists in New Zealand

Semi-structured Interview Schedule for Additional Key Informant Interviews, 2019

1. What is your role with respect to primary health care pharmacy services policy development? How long have you been in the role?

2. Which aspects of policy development have you been involved in over the past few years (e.g., Pharmacy Action Plan, Medicines Management Plan, etc.)?

    a. What have been the drivers for developments in community pharmacy?
    b. What have been the drivers for developing pharmacy roles in general practices and PHOs?

3. What are your expectations for how pharmacy services will look different in the next a) year; b) two - four years?

4. In which communities or for which groups of service users do you expect services to develop more quickly over the next few years?

5. In what ways will such changes benefit the pharmacy profession?

6. In what ways will such changes benefit service users? Which service users do you expect most to benefit?

7. In what ways will such changes benefit the NZ health system?

8. What negative outcomes might arise from the expansion of pharmacist roles?

9. What contextual factors will affect the introduction and development of pharmacy roles in primary health care services? e.g.:

a. Contracts
b. Employer expectations
c. Funding/resourcing
d. Location of practice
e. Infrastructure
f. Recognised role value
g. Mentors/supporters
h. Relationship building
i. National support
j. General practice workforce shortages
k. Pharmacist career pathways
l. Model of practice (substitute/complement/role definition)
m. Size and staffing of community pharmacies (e.g. use of technician roles; any limitations for sole practitioners)
n. Use of technology (e.g. robotics, shared records, e-prescribing)

10. How will proposed changes in the Therapeutic Products Bill impact on primary health care pharmacy developments? Are there any other upcoming legislative or regulatory changes we should be aware of?

11. Are there any other issues relating to the expansion of primary health care pharmacy services that you would like to tell us about?

Thank you very much for your time.

B. Case study interview schedules

See following pages.

EXPANDING THE ROLE OF PRIMARY HEALTH CARE PHARMACISTS IN NEW ZEALAND

INTERVIEW SCHEDULES

Practice pharmacists

The following schedule acts as a guide to maintain and prompt interview flow. Question order may be changed.

When interviewing pharmacist, consider whether they operate in multiple practice/ workplaces and the differences between these.

1. **Tell us about your current role**
   - What would a typical day look like?
     - What does the role involve? (e.g. audit; updating records from hospital discharge summaries; medication review; prescribing; face-to-face contact with patients – individually or in groups; liaison with other pharmacists and other health or social service providers; medicines advice to other health providers; medicines reconciliation; pre-booked appointments/ consultations)
   - Why/ how did you come to be in this role?
   - How has the role changed/ developed over time? How does it influence the workflow/ work within the practice?
   - How did you go about getting this role? (e.g. advertised, word of mouth, knew the owner/ established relationship) How difficult was it to get?
   - How long have you been in the role?
   - How many years have you practised as a pharmacist?
   - What other pharmacy roles have you had before this? (community, hospital pharmacy, other; duration of each)
     - Are you still working in any other pharmacy roles as well as this one?

2. **Tell us about your workplace(s) and practice**
   - How many contracts/ workplaces?
     - Who employs you? (full time/ part time) How does this influence your role/ integration?
     - How long are your contracts valid for? (yearly/ permanent)
     - What is the difference between your roles in these places/ based on your contracts?
     - How are the workplaces responding to your role/ skills?
     - How does your <u>employer</u>/ <u>contract structure</u>/ <u>population needs</u> influence the services you can provide, how much autonomy do you have to choose what you do?
   - What factors influence the development of your role/ your practice in these workplaces? How?
     - Consider factors inside and outside the workplace (e.g. tertiary care responsiveness/ other practices)
     - Do these same factors support your role development in other workplaces? Why/ why not?
   - What do you think enables roles like yours to work to best effect? What is missing in your workplace?
   - How are patients/ service users referred to you?
     - Self-referral? Referral by another health professional <u>outside</u>/ <u>inside</u> the practice?
     - Booking systems? Role of the receptionists?
     - How does this influence your practice?
   - Availability of/ placement of room/ desk/ office/ computer (resources)
     - Do you have a shared office, what wing are you in (e.g. nursing/ doctor's wing)?
     - Do you have access to electronic resources/ other resources required for your role?
     - Part of a health care home/ other model of practice/ Cornerstone?
     - How do these resources/ factors influence your practice?

- Ability to refer for tests/ read tests/ access lab results etc.

3. Tell us what it was like starting in this workplace?
- Induction
- Support
- Supervision and/or mentoring (who? In the workplace or external?)
- Monitoring and evaluation of role? Need to 'prove' self or role value?
- Expectations vs. reality. Why does this differ?
- How well did other staff understand/ accept your role? How has this changed over time?
- When you first started, how well equipped did you feel to undertake/ perform this role?

4. How did your education and training prepare you for your role?
- Do you have a postgraduate qualification? How has this influenced your role/ career?
- Are you a pharmacist-prescriber or interested in becoming one? Why/why not?
- If the pharmacist trained in their current workplace, how did your workplace support your training?

5. Tell us about the kinds of service users you work with?
- e.g. People with long term conditions; audits for wide or narrow range of conditions
- For those with limited/ no face-to-face contact:
    - Would you like this?
    - Is this needed for your role?
    - How would this change your satisfaction/ service you provide?
    - Why is this not part of your role?
- What factors influence the services you provide them? How?
    - Other health professionals/ receptionist/ other staff
- How do service users respond to your role? What factors influence their views on your role?
    - How you deliver services (e.g. accessibility via telephone/ email/ same day appointments/ length of appointment/ language used)
    - Ability to meet cultural and language needs
    - Health literacy needs
    - Do patients pay for pharmacist consultations? How does this affect service access?
    - Trust and confidence in the pharmacists' role
    - Attitudes of other health professionals/ receptionist/ other staff to the pharmacist's role
    - How well do they understand/ accept your role? How has this changed over time?
- What are the outcomes/ benefits of your role for service users? Please provide examples.
    - Are there specific populations who are more likely to benefit, why/ why not?
    - For Māori, Pacific and low-income service users

6. Tell us about your professional relationships with your co-workers? How does this influence the services you provide?
- Do other colleagues understand what you do?
- How do they make referrals to you? How appropriate are these referrals?
- To what extent would your work involve managing inappropriate referrals/ work at the lower end of your competencies
- How do you communicate about service users, e.g. through practice records; face-to-face
- How is responsibility for patient care/ service delivery managed?
    - How is interprofessional work managed (e.g. if the patient's care needs are complex), can you give an example?
- How do you build the trust your co-workers have in your skills/ role?
    - What influences your autonomy in making care decisions?
    - For pharmacist prescribers: Could you, for example, change prescriptions without talking to the initial prescriber – how does your practice team view this?
- Give me an example of how you were able to use your skill set in a way that no one else could/ your contribution changed the care given.

7. The New Zealand Health Strategy and Pharmacy Action Plan talk about integrated health services/ collaboration between health professionals. What factors enable integration/ collaboration between you and other health providers here?
- What is the team environment like/ what are your interactions with other team members like?
    - To what extent do you feel part of the team? Why/ why not?
    - Formal (e.g. meetings) and informal (e.g. tearoom) relationships
        - Are you included in routine clinical meetings?

    - Have new meetings been set up because of your role/ are meetings more ad hoc?
- Relationships (pre-existing; time to develop)
- Being co-located (including shared access to patient information)
- Local workforce pressures
- Wider system pressures (e.g. hospital pressures/ not recognising role)

8. **Outside of this workplace, what other factors influence the development/ success of your role? How?**
   - Support for your role from other pharmacists or mentors within the pharmacy profession
   - Support for your role by other health professionals or mentors outside of pharmacy
   - Support from PHO/ DHB
   - Support from regional pharmacy networks, local networks, membership organisations/ special interest groups (e.g. CAPA, Pharmaceutical Society)
   - Support from family/ friends
   - Personal drive/ motivation
   - Critical mass/ seeing others do it

9. **What do you think hinders the development of similar roles in other practices or locations in New Zealand? How?**
   - Funding
   - Patch-protection, feelings of competition
   - What else is needed to develop the role to its full potential throughout New Zealand?

10. **How do you think primary health care pharmacy roles will develop in the next 5 to 10 years?**
    - How do you see your own future in primary health care pharmacy?
    - What would need to change to retain you/ that would lead to you leaving primary health care pharmacy?

11. **Has your role changed as a result of the COVID-19 pandemic? How?**
    - How do you expect these changes to continue in the future?
    - Do you think your role might change in the future as a result of changes your practice has made due to COVID-19?

12. **Many practice pharmacists/ pharmacist prescribers must 'prove' themselves to their employer to ensure their continued viability. What data do you collect on your role?**
    - How does this influence your ability to prove your value?

13. **Use ranking cards (see ranking exercise document).**

14. **Is there anyone else you would recommend I talk to about primary health care pharmacy in this area?**

15. **Are there any documents specific to your role that you think could help me understand it? If so, how and where can I access these documents?**
    - Job description

16. **Is there anything else you would like to add?**

17. **Ask to fill in demographic form if not already completed.**

GPs/ NPs or PHC nurses/ Practice manager

The following schedule acts as a guide to maintain and prompt interview flow. Question order may be changed. Participants may not be able to answer all questions.

1. **What is your current professional role?**
   - How long have you been practising professionally?
   - How long have you been working in this location?
   - For nurses, are you prescribers?
   - What previous experience have you had working with a pharmacist?
   - What is your current experience/ relationship with the pharmacist working in this practice?
2. **We are interested in the role of the pharmacist in this practice/location.**
   - How long has this pharmacist role existed here? How did it come about?
     - How important are pre-existing relationships between the pharmacist and others in the practice in creating the pharmacist role?
     - How important is having the right 'fit' of pharmacist i.e. personality, over and above technical competence?
   - Is this the first person in the role here? If not, what is your previous experiences with the role, why was it decided to continue the role?
   - What resources did you need to establish the role? (e.g. funding; office space; computer and access to patient records)
   - Is there a job description for the role? Who was involved in creating that? How closely does the current role match the job description?
   - What induction was provided? How was the role originally introduced into the practice?
   - What ongoing support is provided? (e.g. supervision; external peer group)
   - How well does the pharmacist's training prepare them for their current role?
     - If the pharmacist also trained in this practice, how did your workplace support the pharmacist's training?
   - What do you think enables roles like that of your pharmacist to work to best effect? What is missing in your workplace?
3. **How is the pharmacist employed?**
   - By the practice? By the PHO or DHB? Jointly?
   - Are they employed full time/ part time? How does this influence their role/ integration?
   - Who funds the role?
   - If funded externally: Would this practice continue to fund the role if external funding ceases? Why/ why not?
4. **What does the pharmacist's role involve?**
   - e.g. audit; updating records from hospital discharge summaries; medication review; prescribing; face-to-face contact with patients – individually or in groups; liaison with other pharmacists and other health or social service providers; medicines advice to other health providers; medicines reconciliation; pre-booked appointments/ consultations
   - Has the role changed/ developed over time? Why?
   - Who determines what the pharmacist does day to day?
     - How much direction is there from the practice, PHO or DHB?
     - How much autonomy does the pharmacist have in their role? What influences this?
     - Give me an example of when the pharmacist was able to use their skill set in a way that no one else could/ their contribution changed the care given.
     - For pharmacist prescribers: Can they, for example, independently change prescriptions without talking to the initial prescriber – how is this viewed?
   - How does this role influence the workflow/ work within the practice?
     - e.g. substitution of roles, more time for patient contact, referrals to different health professionals within a practice.
     - How does the pharmacist work/ interface with your role? e.g. supporting your prescribing practice, role in smoking cessation/ weight management.
   - How does this role differ from that of a community pharmacist?
     - How has your relationship with community pharmacy changed because of having a pharmacist on your team?

- What sorts of patients generally use the pharmacist's services?

5. **Can this pharmacist prescribe?**
   - If yes, how do you view this role? What kind of patients are they prescribing for?
     - What benefits does this have for a) patients b) yourself?
     - Do you have any concerns about this aspect of the role? How are they managed?
   - If no, would you support the introduction of a prescribing pharmacist? How would you envisage this role working in your practice?

6. **How does the pharmacist's role support you as a GP/ NP (or PHC nurse)?**
   - Support the work of other practice staff? (e.g. through training, reference material, advice)
   - How does your role support the pharmacist's role?

7. **How do you communicate with the pharmacist about patients, e.g. through practice records; face-to-face discussion; regular meetings**
   - How is responsibility for patient care/ service delivery managed?
     - How is interprofessional work managed (e.g. if the patient's care needs are complex), can you give an example?

8. **How did staff initially respond to the pharmacist's role?**
   - How has this changed over time?
     - What factors resulted in this change in response/ understanding?
     - How is trust built between the pharmacist and co-workers?
   - How well do they understand the pharmacist's role?

9. **What kind of patients does the pharmacist work with?**
   - e.g. people with long term conditions; audits for wide or narrow range of conditions

10. **How are patients referred to the pharmacist?**
    - Booking systems, referrals, word of mouth? Any referrals from outside the practice?
    - Do all providers in the practice refer patients, why/ why not?
    - Who manages referrals? What is the impact on reception or other staff?
    - How are patients advised about the existence of the role?

11. **How have service users responded to the pharmacist's role?**
    - What factors influence the services pharmacists provide them? How?
      - Other health professionals/ receptionist/ other staff
    - What factors influence service user views on the pharmacist's role?
      - How the service is delivered (e.g. accessibility via telephone/ email/ same day appointments/ length of appointment/ language used)
      - Ability to meet cultural and language needs
      - Health literacy needs
      - Do service users pay for consultations with a pharmacist? How does this affect access to the service?
      - Trust and confidence in the pharmacists' role
      - Other health professionals/ receptionist/ other staff
      - How well do patients understand/ accept the role? How has this changed over time?
    - What are the outcomes/ benefits of the pharmacist's role for service users? Please provide examples.
      - Are there specific populations who are more likely to benefit, why/ why not?
      - For Māori, Pacific and low-income service users
    - Have service users had any involvement in the co-design of these roles?

12. **The New Zealand Health Strategy and Pharmacy Action Plan talk about integrated health services/ collaboration between health professionals. What factors enable integration/ collaboration between the pharmacist and other health providers here?**
    - What is the team environment like?
      - To what extent is the pharmacist part of the team? Why/ why not?
      - How is the pharmacist included as part of the team e.g. through regular meetings?
    - Relationships (pre-existing; time to develop)
    - Being co-located (including shared access to patient information)
    - Local workforce pressures
    - Wider system pressures (e.g. hospital pressures/ not recognising role)

13. **What do you think hinders the development of similar roles in other practices or locations in New Zealand?**
    - Funding
    - Patch-protection, feelings of competition
    - What else is needed to develop the role to its full potential throughout New Zealand?
14. **How do you think primary health care pharmacy roles will develop in the next 5 to 10 years?**
15. **Has the pharmacist's role changed as a result of the COVID-19 pandemic?**
    - How do you expect these changes to continue in the future?
    - Do you think their role might change in the future as a result of changes you've had to make due to COVID-19?
16. **Many practice pharmacists/ pharmacist prescribers must 'prove' themselves to their employer to ensure their continued viability. What data does your pharmacist collect on their role?**
    - How does this influence their future viability?
17. **Use ranking cards (see ranking exercise document).**
18. **Is there anyone else you would recommend I talk to about primary health care pharmacy in this area?**
19. **Is there anything else you would like to add?**
20. **Ask to fill in demographic form if not already completed.**

Reception staff

The following schedule acts as a guide to maintain and prompt interview flow. Question order may be changed.

1. **What is your current professional role?**
    - How long have you been practising professionally?
    - How long have you been working in this location?
    - What previous experience have you had working with a pharmacist?
    - What is your current experience/ relationship with the pharmacist working in this practice?
2. **We are interested in the role of the pharmacist in this practice/ location.**
    - How long has this pharmacist role existed here?
    - What is the pharmacist's role in this practice?
    - What does the pharmacist add to the practice? (How does this benefit both staff and patients?)
    - How does this role influence the workflow/ work within the practice?
    - How does this role differ from that of a community pharmacist?
3. **How does your role support the pharmacist's?**
    - e.g. managing bookings; any assistance with records; facilitating calls to/ from patients
    - How much time does this involve for you daily or weekly?
    - Have you needed any additional resources to manage this work?
    - How do bookings get made for the pharmacist, e.g. referrals from others in the practice, external referrals, patient-led referrals?
    - What sorts of patients generally use the pharmacist's services?
4. **What feedback have you had from patients about the pharmacist's role? Can you provide examples [particularly for when they were willing/ reticent to see the pharmacist]?**
    - How is the pharmacist's role promoted in your practice?
    - Do you ever need to explain the pharmacist role to service users?
        - If so, how do you explain the pharmacist's role to service users?
        - If not, why?
    - How well do they understand and accept the role?
        - What factors influence their views on the pharmacist's role?
    - Have patient views about the pharmacist's role changed over time?
        - What causes this change in understanding/ acceptance?
    - Do service users pay for consultations with a pharmacist? How do you think this affects access to the service? Have you had any feedback from service users about this?

- What groups in particular respond well/ poorly to the pharmacist's role? Why?

5. **What do you think has supported the development of the pharmacist's role here?**
   - What enables the role to work well?
   - What could be done to improve the way this role contributes in this practice?
   - What feedback have you had from others in your workplace about the pharmacist's role?
     - How do others in the practice see/ use the pharmacist's role?
     - When the role was first introduced, how did individuals react to it/ how was it promoted internally?
     - What factors influence others' views of the pharmacist's role?

6. **Use ranking cards (see ranking exercise document).**

7. **Is there anything else you would like to add?**

8. **Ask to fill in demographic form if not already completed.**

DHB pharmacy leads

The following schedule acts as a guide to maintain and prompt interview flow. Question order may be changed.

1. **What is your current professional role?**
   - How long have you been working in this location?
   - What is your professional background – are you a pharmacist?
   - Outside of this role, what is your experience/ relationship with pharmacy?

2. **We are interested in the roles of pharmacists in primary health care, both in community pharmacy and in roles in general practices or across a PHO.**
   - First of all, do you have a pharmacy strategy in this DHB? If so, how did it develop? If not, are you planning to develop one in future?
     - Where did support for this strategy/ the creation of new pharmacy roles come from?
   - Health alliances in your region – How is pharmacy involved in these? (particularly PHC)
   - What outcomes are you wanting to achieve from pharmacy services?

3. **Thinking first about community pharmacy services, how have these been developing in this DHB in the last 5 to 10 years?**
   - e.g. CPAMS, vaccinations, MUR
   - Are all services available at all community pharmacies? Why or why not?
   - What features of particular community pharmacies make them more/ less likely to offer extended services?

4. **Is there a community pharmacy group in this region?**
   - How did that come about?
   - How has that influenced the development of services in community pharmacy here?
   - Do you have any locally-commissioned services under the ICPSA contract? What and why/why not?
   - If no CPG: How do you manage relationships with community pharmacies in this area?

5. **Now focusing on other primary health care pharmacy roles: how have these been developing in this DHB?**
   - e.g. pharmacists in PHOs or general practices
   - How did this come about?
   - How many practice pharmacists are there in your DHB?
   - How many practices do these pharmacists work over?
     - Is this all the practices in the area?
     - If only some, how were these chosen and are there plans to extend coverage (or what hinders extension)?
   - Did the creation of these roles differ between practices, if so, how?
     - e.g. were they limited in services they could offer in one practice compared to another
     - Were service users involved in the co-design of these roles?
     - Do service users have continuing involvement in these matters?
   - What kinds of services are these pharmacists offering? Who defines what they do?
   - What outcomes do these roles provide:
     - for service users?

    - for other health providers? (e.g. support for workforce pressures; support with prescribing quality/ medicine appropriateness)
    - for pharmacists?
- How have national initiatives/ policies (e.g. the Pharmacy Action Plan) or Ministry/ TAS directives influenced development of primary health care pharmacy roles in this DHB?

6. **How have primary health care pharmacy roles been received by:**
   - Other health professionals (especially GPs; hospital staff; also community pharmacists)
   - Services users
   - How has this changed over time?
   - Do you think they are a good idea? Why/ why not?
   - Are there any other things could they be doing?

7. **What factors have influenced the introduction/ implementation of primary health care pharmacy roles in this DHB? How?**
   - Funding? Who funds these roles?
     - Availability of funding for education/ formal training pathways
     - Funding of the role/ length of contract
   - Contracts – short or long term?
   - Support from within pharmacy
   - Support from the wider health system
   - Involvement of the DHB/ PHO in facilitating employment
   - Personal drive/ motivation of pharmacists to develop new roles
   - Critical mass/ seeing others do it (either other DHBs, or other practices/ PHOs)
   - Sense of competition between community pharmacy/ PHC practice pharmacists OR by general practice

8. **What factors have influenced the continued development/ sustainability of primary health care pharmacy roles in this DHB? How?**
   - Continuing funding
   - Support from within pharmacy/ the wider health system (query role of CPG)
   - How sustainable is the practice pharmacist role? What would make it sustainable?

9. **How does the introduction/ implementation of practice pharmacist roles fit in with changes in pharmacy more widely (e.g. community pharmacy, hospital pharmacy)?**

10. **Monitoring/ evaluation of the practice pharmacist role is often discussed. What data does this DHB require of their practice pharmacists?**
    - What does this data show about the value of these roles? How does this influence decisions to keep these roles?
    - Are any reports publicly available?

11. **What hinders the development of similar roles in other practices or locations in New Zealand? Why?**
    - What else is needed to develop pharmacy in primary health care to its full potential throughout New Zealand?

12. **How do you think primary health care pharmacy roles will develop in the next 5 to 10 years?**
    - In your region
    - Nationally
    - How do these roles fit with wider health system/ population health changes in your region?

13. **Use ranking cards (see ranking exercise document).**

14. **Are there any documents specific to the practice pharmacist role in your DHB that you think could help me understand how this role is developing? If so, how and where can I access these documents?**
    - Job description
    - DHB policy documents
    - Monitoring/evaluation reports in general/ for each practice pharmacist (Q10)

15. **Is there anyone else you would recommend I talk to about primary health care pharmacy in this area?**
16. **Is there anything else you would like to add?**
17. **Ask to fill in demographic form if not already completed.**

PHO pharmacy leads

The following schedule acts as a guide to maintain and prompt interview flow. Question order may be changed.

1. **What is your current professional role?**
   - How long have you been working in this location?
   - What is your professional background – are you a pharmacist?
   - Outside of this role, what is your experience/ relationship with pharmacy?
2. **Please tell me about pharmacist roles in this PHO.**
   - e.g. pharmacists in PHO or general practices
   - How did this come about?
   - How many practice pharmacists are there in your PHO?
   - How many practices do these pharmacists work over?
     - Is this all the practices in the area?
     - If only some, how were these chosen and are there plans to extend coverage (or what hinders extension)?
   - Did the creation of these roles differ between practices, if so, how?
     - e.g. were they limited in services they could offer in one practice compared to another
     - Were service users involved in the co-design of these roles?
     - Do service users have continuing involvement in these matters?
   - Are any of your practices Health Care Homes? How has this influenced the development of pharmacy roles?
   - Who funds these roles? [If the PHO, could we have a copy of the pharmacist's contract and job description?]
   - What kinds of services are these pharmacists offering? Who defines what they do?
   - What outcomes do you expect from these roles:
     - for service users?
     - for other health providers? (e.g. support for workforce pressures; support with prescribing quality/ medicine appropriateness)
     - for pharmacists?
   - How have national initiatives/ policies (e.g. the Pharmacy Action Plan) or Ministry/ TAS directives influenced development of primary health care pharmacy roles in this DHB?
   - Is there a pharmacy strategy in this DHB/ PHO? How has this influenced development of primary health care pharmacy roles in this DHB/ PHO?
   - Is there a community pharmacy group in this DHB? Were they involved in the development of primary health care pharmacy roles in this DHB/ PHO?
3. **How have primary health care pharmacy roles been received by:**
   - other health professionals (especially GPs; also community pharmacists)
   - services users
   - How has this changed over time?
   - Do you think they are a good idea? Why/ why not?
   - What other things could they be doing?
4. **What factors have influenced the introduction/ implementation of primary health care pharmacy roles in this PHO/ in various practices? How?**
   - Health alliances in your region – How is pharmacy involved in these?
   - Funding? Who funds these roles?
   - Support from within pharmacy/ the wider health system
   - Contracts – short or long-term?
   - Involvement of the DHB/ PHO in facilitating employment
   - Personal drive/ motivation of pharmacists to develop new roles
   - Critical mass/ seeing others do it (either other DHBs, or other practices/ PHOs)

- Sense of competition between community pharmacy/ PHC practice pharmacists OR by general practice

5. **What factors have influenced the continued development/ sustainability of primary health care pharmacy roles in this PHO/ in various practices? How?**
   - Continuing funding
   - Support from within pharmacy/ the wider health system
   - Contracts – whether these are short or long term
   - How sustainable is the practice pharmacist role? What would make it sustainable?
6. **Use ranking cards (see ranking exercise document).**
7. **What hinders the development of similar roles in other practices or locations in New Zealand?**
   - What else is needed to develop pharmacy in primary health care to its full potential throughout New Zealand?
8. **How do you think primary health care pharmacy roles will develop in the next 5 to 10 years?**
   - In your PHO
   - In your DHB
   - Nationally
   - How do these roles fit with wider health system/ population health changes in your region?
9. **Monitoring/ evaluation of the practice pharmacist role is often discussed. What data does this PHO require of their practice pharmacists?**
   - How does this influence their long-term viability?
10. **How does the introduction/ implementation of practice pharmacist roles fit in with wider changes in pharmacy? How does it fit with your PHO's interactions with community pharmacy?**
11. **Are there any documents specific to the practice pharmacist role in your PHO that you think could help me understand how this role is developing? If so, how and where can I access these documents?**
    - Job description
    - DHB policy documents
    - Monitoring/evaluation reports in general/ for each practice pharmacist (Q9)
12. **Is there anyone else you would recommend I talk to about primary health care pharmacy in this area?**
13. **Is there anything else you would like to add?**
14. **Ask to fill in demographic form if not already completed.**

Service users

The following schedule acts as a guide to maintain and prompt interview flow. Question order may be changed.

Some questions may need to be amended if the pharmacist does not work in a practice.

I understand you have received services from the pharmacist at _______ general practice [or possibly at home/ other primary health care workplace]. We'd like to learn about your experiences with those services, including the way they're offered and whether you'd like anything changed.

1. **Can you tell me how you came to be using these pharmacist services?**
   - How did you find out about the service?
   - Who referred you to the service?
   - How did you feel when you were first offered an appointment with/ referred to the pharmacist? What did you expect?
   - How long ago did you first use this service?
   - How is this role promoted in your practice?
2. **Tell me about the services your pharmacist has offered you? What do they do?**
   - Around your medication
     - Education about their purpose/how they work
     - Changing your medicines (e.g. dose; stopping a medication)

    - o Advice on when/ how you take your medicines/ techniques to remember to take them/ how to adjust doses
    - o Have they provided any written resources? (e.g. information; record charts)
    - o Is the pharmacist able to prescribe medication for you?
- Have they offered broader education about a health condition?
- Have they referred you to any other health or social services (including community pharmacy)?
- Before you saw this pharmacist, what did you think a pharmacist's role is? How would you describe what they do?
- How has your understanding of pharmacist roles changed from using this service?

3. **How does the pharmacist meet your individual needs?**
    - How do they work differently from other health professionals? E.g. doctors, nurses
    - What does the pharmacist add to the information/ advice/ support you get from other staff in the practice?
    - What about cultural needs?

4. **How have these services helped you?**
    - e.g. Ability to check results, follow up with you, offer recommendations, refer externally
    - How do they work with others in your practice?
        - o e.g. do you think they talk with your GP/ practice nurse about your care? Are you OK with this?
        - o If they work part time, how does this influence the services they offer you/ interactions with others in your practice e.g. do you get followed up by another health provider instead?
    - Have they needed to speak with community pharmacy/ other health providers outside the practice? How does this work?
        - o Is this alright with you?

5. **How have you met with the pharmacist?**
    - Face to face – at a general practice; at your home or elsewhere
    - By phone
    - By videoconference
    - By email
    - Other
    - I don't meet with the pharmacist, but I hear about their recommendations from my GP/ nurse/ other
    - How do you prefer to meet? Why?

6. **How often do you have contact with the pharmacist? How often do they get in contact with you?**

7. **How do you organise an appointment with the pharmacist?**
    - Is there a booking system? How does this work? (e.g. phone receptionist; available through 'Manage My Health'; phone pharmacist directly)
    - How quickly can you get an appointment?
    - Do you have to be referred by the doctor or can you ask for an appointment directly?

8. **Is there a charge for this service?**
    - If yes, what do you think about the amount you pay?
    - If no, would it impact on your use of the service if you did have to pay?

9. **Use ranking cards (see ranking exercise document).**

10. **Is there anything you'd like to change about the way pharmacy services are offered at this practice?**
    - Are there any other services you'd like the pharmacist to provide?
    - Is there anything else in general that a pharmacist working outside a pharmacy should be doing to help people manage their health?

11. **What is the difference between the pharmacist services you've been talking about and the services you'd get at a community pharmacy/chemist shop.**
    - Do you also use any services at a community pharmacy? Could you tell me about the sorts of services you receive there?
    - Would you like the kind of pharmacy service you've received here to be offered at a community pharmacy too? Why/why not?

- Does the pharmacist working in this practice/ you've been talking about interact with your local community pharmacy? How?

12. Is there anything else you would like to add?

13. Ask to fill in demographic form if not already completed.

Ranking exercises

EXPANDING THE ROLE OF PRIMARY HEALTH CARE PHARMACISTS IN NEW ZEALAND

RANKING EXERCISE

Time permitting, a ranking exercise to summarise the interview and get views about the most important contexts and mechanisms that are operating.

Pharmacists

Rules

1. Provide set of cards with topics (below)
2. Ask: Thinking back to when you began in this role, what were the 3 most important things for supporting the establishment of your role?
   - When selected, ask for further explanation of why these were chosen.
   - Do you think these 3 would apply to all pharmacists in similar roles? Why/ why not?
3. Provide full set of cards again
4. Ask: Now thinking about your current work situation, what are the 3 most important things for maximising the effectiveness of your role?
   - When selected, ask for further explanation of why these were chosen.
   - Do you think these 3 would apply to all pharmacists in similar roles? Why/ why not?

Topics

- Who contracts and funds the service
- Physical location of the pharmacist (i.e. whether you work off site, whether you've got your own permanent office, where the office is situated)
- Amount of time regularly spent in a practice
- Technology
- Access to patient information and ability to contribute to shared patient records
- Trusting relationships with other practice staff
- Existing kaupapa for interprofessional working/ non-hierarchal inclusive staff approach
- Support with appointment bookings
- Other practice staff respecting and valuing the pharmacist's role
- Other practice staff utilising the pharmacist's role appropriately
- Active promotion of the pharmacist's services to service users
- Service users understanding and trusting the pharmacist's role

- Professional support networks for the pharmacist
- Responsiveness of other health professionals outside your workplace to your decision-making or referrals
- Pre-existing relationships with practice staff
- one “blank” option for them to add something else

GPs/ Nurses/ Practice managers/ Receptionists

Rules

1. Provide set of cards with topics (below)
2. Ask: Thinking back to when the pharmacist role was established in this practice, what were the 3 most important things for supporting the establishment of the role?
   - When selected, ask for further explanation of why these were chosen.
   - Do you think these 3 would apply to all pharmacists in similar roles? Why/ why not?
3. Provide full set of cards again
4. Ask: Now thinking about the current way the pharmacist works here, what are the 3 most important things for maximising the effectiveness of this role?
   - When selected, ask for further explanation of why these were chosen.
   - Do you think these 3 would apply to all pharmacists in similar roles? Why/ why not?

Topics

- Who contracts and funds the service
- Physical location of the pharmacist (i.e. whether you work off site, whether you’ve got your own permanent office, where the office is situated)
- Being a health care home/ other model of practice/ Cornerstone accredited
- Amount of time regularly spent in a practice
- Technology
- Access to patient information and ability to contribute to shared patient records
- Trusting relationships with other practice staff
- Existing kaupapa for interprofessional working/ non-hierarchal inclusive staff approach
- Support with appointment bookings
- Other practice staff respecting and valuing the pharmacist’s role
- Other practice staff utilising the pharmacist’s role appropriately
- Active promotion of the pharmacist’s services to service users
- Service users understanding and trusting the pharmacist’s role
- Professional support networks for the pharmacist
- Responsiveness of other health professionals outside your workplace to your decision-making or referrals
- Pre-existing relationships with practice staff
- one “blank” option for them to add something else

DHB leads

Rules

1. Provide set of cards with topics (below)
2. Ask: Thinking back to the time when the pharmacist’s role was first being developed in general practices in this area, what were the 3 most important things for supporting the establishment of the role?
   - When selected, ask for further explanation of why these were chosen.
   - Do you think these 3 would apply to all pharmacists in similar roles? Why/ why not?
   - Do these 3 factors still apply now to new pharmacist roles? Why/ why not?
3. Provide full set of cards again
4. Ask: Now thinking about the present time and the way pharmacists are working in general practice, what are the 3 most important things for maximising the effectiveness of this role?
   - When selected, ask for further explanation of why these were chosen.

    - Do you think these 3 would apply to all pharmacists in similar roles? Why/ why not?

Topics

- Who contracts and funds the service
- Working at one or multiple practices
- Trusting relationships with other practice staff
- Access to patient information
- Being part of a Health Care Home
- Existing kaupapa for interprofessional working/ non-hierarchal inclusive staff approach
- Other practice staff respecting and valuing the pharmacist's role
- Other practice staff utilising the pharmacist's role appropriately
- Service users understanding and trusting the pharmacist's role
- Professional support networks for the pharmacist
    - Within the pharmacy profession?
    - Support from other health professionals, mentors outside of pharmacy
    - Support from PHO/ DHB
    - Support from regional pharmacy networks, local networks, membership organisations/ special interest groups (e.g. CAPA, Pharmaceutical Society)
- Active promotion to service users (what does this look like here?)
- one "blank" option for them to add something else

PHO leads

Rules

1. Provide set of cards with topics (below)
2. Ask: Thinking back to the time when the pharmacist's role was first being developed in general practices in this area, what were the 3 most important things for supporting the establishment of the role?
    - When selected, ask for further explanation of why these were chosen
    - Do you think these 3 would apply to all pharmacists in similar roles? Why/why not?
    - Do these 3 factors still apply now to new pharmacist roles? Why/ why not?
3. Provide full set of cards again
4. Ask: Now thinking about the present time and the way pharmacists are working in general practice, what are the 3 most important things for maximising the effectiveness of this role?
    - When selected, ask for further explanation of why these were chosen
    - Do you think these 3 would apply to all pharmacists in similar roles? Why/why not?

Topics

- Practice resourcing and support (e.g. office space; assistance with booking)
- Who contracts and funds the service
- Physical location of the pharmacist (i.e. whether they work off site, whether they've got their own permanent office, where the office is situated)
- Amount of time regularly spent in a practice
- Access to patient information
- Being part of a Health Care Home
- Technology (what)
- Trusting relationships with other practice staff
- Existing kaupapa for interprofessional working/ non-hierarchal inclusive staff approach
- Other practice staff respecting and valuing the pharmacist's role
- Other practice staff utilising the pharmacist's role appropriately
- Service users understanding and trusting the pharmacist's role
- Professional support networks for the pharmacist

    - Within the pharmacy profession?
    - Other health professional support, mentors outside of pharmacy
    - Support from PHO/ DHB
    - Support from regional pharmacy networks, local networks, membership organisations/ special interest groups (e.g. CAPA, Pharmaceutical Society)
- Active promotion to service users (what does this look like here?)
- one "blank" option for them to add something else

Service users

Rules

1. Provide set of cards with topics (below)
2. Ask: Thinking about your pharmacist's role, what are the 3 most important things for you around how you receive these services?
    - When selected, ask for further explanation of why these were chosen
    - Do you think these 3 would apply to all pharmacists in similar roles? Why/why not?
    - Have these factors changed at all as you've got to know the pharmacist?

Topics

- Convenience of location/ other location factors
- When the service is available (i.e. days and times)
- Whether or not a referral is needed
- Whether or not an appointment is needed
- Privacy and confidentiality
- Relationship with the pharmacist
    - What is important for a good relationship?
    - What influences your trust in the pharmacist?
    - What makes you feel 'known' by the pharmacist?
- Communication between the pharmacist and your doctor and other health care professionals
    - What sort of relationship do the other members of the practice/ health care team have with your pharmacist? How well do they fit in the team?
- Involving you in decisions about your health and care
- Involving your family/whānau if you want this
- Respect for your culture and language
- one "blank" option for them to add something else
